# Impact of fucosyltransferase 1-mediated epidermal blood group antigen H on anti-inflammatory response in atopic dermatitis

**DOI:** 10.3389/fimmu.2024.1365430

**Published:** 2024-05-22

**Authors:** Na Li, Jang-Hee Oh, Joong Heon Suh, Seon-Pil Jin, Dong Hun Lee, Youngae Lee, Jin Ho Chung

**Affiliations:** ^1^ Department of Dermatology, Seoul National University College of Medicine, Seoul, Republic of Korea; ^2^ Institute of Human-Environment Interface Biology, Medical Research Center, Seoul National University College of Medicine, Seoul, Republic of Korea; ^3^ Laboratory of Cutaneous Aging Research, Biomedical Research Institute, Seoul National University Hospital, Seoul, Republic of Korea; ^4^ Department of Biomedical Sciences, Seoul National University Graduate School, Seoul, Republic of Korea; ^5^ Institute on Aging, Seoul National University, Seoul, Republic of Korea

**Keywords:** ABH antigens, fucosyltransferase 1, atopic dermatitis, cytokines, chemokines

## Abstract

The presence of the blood group H2 antigen on the membrane of red blood cells determines blood type O in individuals and this H2 antigen serves as a precursor to the A and B antigens expressed in blood types A and B, respectively. However, the specific involvement of ABH antigens in skin diseases is unknown. Therefore, we aim to investigate the expression of ABH antigens in skin tissue of patients with atopic dermatitis (AD) and MC903-induced AD-like mice. We demonstrated that the expression of ABH antigen is primarily located in the granular and horny layers of the skin in healthy control individuals. However, in patients with AD, the expression of the ABH antigen was absent or diminished in these layers, while the H2 antigen expression increased in the spinous layers of the affected skin lesions. Then, we investigated the biological function of blood group H antigen mediated by fucosyltransferase 1 (Fut1) in the skin, utilizing an AD mouse model induced by MC903 in wild-type (WT) and *Fut1*-knockout mice. After the application of MC903, Fut1-deficient mice, with no H2 antigen expression on their skin, exhibited more severe clinical signs, increased ear swelling, and elevated serum IgE levels compared with those of WT mice. Additionally, the MC903-induced thickening of both the epidermis and dermis was more pronounced in Fut1-deficient mice than that in WT mice. Furthermore, Fut1-deficient mice showed a significantly higher production of interleukin-4 (IL-4) and IL-6 in skin lesions compared with that of their WT counterparts. The expression of chemokines, particularly *Ccl2* and *Ccl8*, was notably higher in Fut1-deficient mice compared with those of WT mice. The infiltration of CD4^+^ T cells, eosinophils, and mast cells into the lesional skin was significantly elevated in Fut1-deficient mice compared with that in WT mice. These findings demonstrate the protective role of H2 antigen expression against AD-like inflammation and highlight its potential therapeutic impact on AD through the regulation of blood group antigens.

## Introduction

Atopic dermatitis (AD) is a multifaceted and chronic inflammatory skin disease, representing one of the most prevalent skin disorders (1). Current estimates indicate that 10–20% of children and 3% of adults in developed countries are affected by this condition (1–4). AD represents a highly heterogenous clinical and molecular phenotypes (4–6). While the pathogenesis of AD is not fully understood, the disorder is attributed to a combination of genetic and environmental factors, microbial imbalance, immune dysregulation, and abnormalities in the skin barrier (6–9). The amplification of skin inflammation in AD is primarily associated with a T helper-2 (Th2) immune response, characterized by high expression levels of interleukin (IL)-4 and IL-5 (10, 11). The lesional skin of patients with AD exhibited T-cell infiltration, primarily characterized by CD4 expression (1, 12). IL-4 from Th2 cells promotes class switching to immunoglobulin E (IgE) in B cells, leading to the production of antigen-specific IgE (1, 13, 14). Eosinophils and mast cells infiltrate the skin in cases of AD, and their presence was correlated with the severity of the disease (15, 16). Consequently, these immune cells are regarded as potential effector cells due to their production of inflammatory molecules associated with AD (17–19). Moreover, epidermal keratinocytes highly express C-C motif chemokine ligand (CCL) 2 and CCL8 (20, 21) in the skin of patients with AD. CCL2 signaling recruits immune cell infiltration and elicits itch behavior in a mouse model of allergic contact dermatitis (22, 23). CCL8 exhibits chemotactic properties, attracting activated and highly differentiated Th2 cells to the skin during chronic allergic inflammation (24).

The weakening of the skin barrier promotes inflammation and T cell infiltration, subsequently compromising barrier function, intensifying itching, and facilitating microbial dysbiosis, such as colonization with *Staphylococcus aureus* (1, 25, 26). Under inflammatory conditions, keratinocytes produce the epidermal alarmins, such as thymic stromal lymphopoietin (TSLP), leading to tissue damage and driving the recruitment of type 2 inflammatory cells (1, 27–29).

Fucosyltransferase-1 (FUT1) is pivotal in the synthesizing the blood group H2 antigen, a critical determinant for blood type O. This H2 antigen is indispensable for the subsequent formation of A and B antigens, thereby determining blood types A and B, respectively (30, 31). While it is established that ABH antigens determine individual blood types through their expression on the erythrocyte membrane (32), our prior research, along with other studies, have reported that these antigens are predominantly distributed in various tissues. Notably, they are concentrated in regions in direct contact with the external environment, such as the skin, intestines, oral cavity, and liver (33–36). Additional studies have revealed that the expression of FUT1 is restricted to a limited extent within epithelial cells (37–39). The association between the expression of ABH antigens and inflammatory responses, including those induced by UV irradiation, breast and ovarian cancer, respiratory diseases, and pathogen infections, has been explored (33, 39–43). Despite this, the specific involvement of ABH antigens in skin diseases has remained largely elusive. This study sheds light on the anti-inflammatory role of ABH antigens, especially the H2 antigen, within the context of MC903-induced AD-like inflammation in mouse model.

## Materials and methods

### Antibodies and reagents

Antibodies against A (Z2B-1), B (Z5H-2), H2 (BRIC231), FUT1 (G-13), Filaggrin (N-20), and β-Actin (C4) were purchased from Santa Cruz Biotechnology Inc. (Santa Cruz, CA, USA). FUT1 antibody (17956-1) was purchased from Proteintech (Chicago, IL, USA). Antibodies against CD4 (4SM95) and F2RL1 were purchased from Invitrogen (Carlsbad, CA, USA). Eosinophil antibody (BMK-13) was purchased from Novus Biologicals (Littleton, CO, USA). Antibodies against Loricrin (Poly19051) and IL-4 (11B11) were purchased from BioLegend (San Diego, CA, USA). Keratin 10 antibody (EP1607IHCY) was purchased from Abcam (Waltham, Massachusetts, USA). Mcpt6 antibody (MAB3736) was purchased from R&D systems (Minneapolis, MN, USA). MC903 was purchased from Tocris (Bristol, UK). Toluidine blue solution was obtained from Sigma-Aldrich (St. Louis, MO, USA).

### Patients and specimens

Skin tissue samples included 39 patients diagnosed with AD (27 males and 12 females, age range = 19 to 46 years) and 66 healthy controls (10 males and 56 females, age range = 20 to 81 years). Informed consent was obtained from all participants before their involvement, and the study received ethical approval from the Institutional Review Board (IRB No. C-1312-084-543) of Seoul National University Hospital. The study adhered to the principles outlined in the Declaration of Helsinki. Patients with AD who had concurrent acute illnesses were excluded from the study.

### Mice

Fut1-deficient mice (B6.129-Fut1tm1Sdo/J) and wild-type (WT) C57BL/6 mice were obtained from Jackson Laboratory (Bar Harbor, ME, USA). They were housed in a pathogen-free barrier facility. Male mice aged six weeks were used in this study, which was approved by the Animal Care and Use Committee (IACUC No. 20-0112-S1A0) of Seoul National University Hospital. The study was performed in accordance with the National Institutes of Health Guide for the Care and Use of Laboratory Animals.

### Immunohistochemical staining

Briefly, 4 µm sections from the formalin-fixed paraffin-embedded skin specimens of lesional and nonlesional skin from patients with AD and healthy skin were dewaxed in xylene, rehydrated in graded alcohol, and washed with distilled water. For frozen tissue specimens, the sections were fixed in acetone for 5 min at -20°C. Endogenous peroxidase activity was quenched by a 6-min incubation with a 3% hydrogen peroxide solution. Following a 30-min block with the blocking solution from the SPlink HRP Broad Bulk Kit (Golden Bridge International, Mukilteo, WA, USA), the specimens were incubated with monoclonal primary antibodies against A, B, or H2 antigen in a humidified chamber for 18 h at 4°C. Subsequently, they were incubated with biotinylated secondary antibodies for 15 min and then with streptavidin-horseradish peroxidase conjugate for 15 min at 25°C (Golden Bridge International). Immunostaining signals were developed using 3-amino-9-ethylcarbazole (Golden Bridge International) for 3 to 5 min, depending on the types of antibodies, and the slides were mounted with Paramount medium (DAKO, Carpinteria, CA, USA). Images were captured using a Leica DFC280 camera (Leica, Heerbrugg, Switzerland) coupled with an Olympus BX51 microscope (Olympus, Tokyo, Japan). Semiquantitative evaluation of the images was independently performed by three dermatologists on a scale of 0–4. Standard images, ranging from of grade 0 (no staining) to grade 4 (strongest staining), were selected from all staining images. The grade values of each group were statistically analyzed using the Mann–Whitney U Test for comparison between healthy skin and lesional or nonlesional skin from patients with AD. The Wilcoxon signed*-*rank test was applied for paired samples of lesional and nonlesional skin from the same patients with AD.

### MC903-induced AD-like skin inflammation in mice

In the MC903-induced AD-like model, 1.0 nmol of MC903 in 25 μL ethanol was topically applied on both ears for 12 d (44). The ear thickness was measured daily using a digital caliper (Mitutoyo Corp., Tokyo, Japan). At the end of experiment on day 13, the ear skin was snap-frozen in liquid nitrogen for RNA and protein isolation, and the ear was embedded in Optimal Cutting Temperature compounds (Invitrogen) to prepare frozen sections.

### Histology, immunofluorescence analysis, and toluidine blue staining

Skin was fixed in 4% paraformaldehyde at 4°C overnight, paraffin-embedded, sectioned into 4 µm, and stained with hematoxylin and eosin. Images were acquired using a Nikon ECLIPSE Ci-L microscope (Nikon Instruments Inc., USA). Epidermal and dermal thicknesses were measured using ImageJ 1.53e software (NIH). For immunofluorescence, skin sections were blocked in pre-blocking solution (GBI Labs, Bothell, WA, USA) and stained with the indicated primary antibodies at 4°C overnight. After washing, the sections were incubated with an Alexa Fluor 488-conjugated secondary antibody at 25°C for 1 h and stained with 4′-6-diamidino-2-phenylindole dihydrochloride (R37606; Thermo Fisher Scientific, Waltham, MA, USA) at 25°C for 5 min. Images were acquired using a confocal microscope (Leica STED CW; Leica Microsystems, Mannheim, Germany). For the detection of mast cells, ear sections were stained with 0.5% toluidine blue at 25°C for 15 min and then washed three times in PBS. Images were acquired using a Nikon ECLIPSE Ci-L microscope. The number of immune cells was counted every five fields (40× objective), and the average was calculated.

### ELISA

The level of *in vivo* cytokine and IgE production in mice was measured using ELISA according to the manufacturer’s protocol (BioLegend).

### Quantitative PCR

Total RNA was isolated from skin and cells using RNAiso Plus (Takara Bio Inc., Shiga, Japan) according to the manufacturer’s instructions. Subsequently, 1.0 μg of the total RNA was converted to cDNA using a First-Strand cDNA Synthesis Kit (Thermo Fisher Scientific), according to the manufacturer’s instructions. Quantitative RT-PCR was performed using AccuPower^®^ 2X GreenStar™ qPCR Master Mix (Bioneer, Daejeon, Korea), and an ABI 7500 Real-Time PCR instrument (ABI, Indianapolis, IN, USA) was used to measure relative mRNA expression levels. Each sample was analyzed in duplicate, and the expression levels were normalized to those of the housekeeping gene, *36B4*.

### Flow cytometry

Single-cell suspensions from the ear-draining lymph nodes (LNs) were pre-treated with an anti-CD16/32 antibody (BD Biosciences, San Jose, CA, USA) prior to staining with the specified antibodies. Intracellular staining was conducted using the BD Cytofix/Cytoperm™ Fixation/Permeabilization Kit (BD Biosciences) in accordance with the manufacturer’s protocol. Samples were assessed with the BD FACSCanto™ II (BD Biosciences), and data were analyzed using FlowJo software (BD Biosciences).

### Western blot analysis

Tissue lysates were prepared in 1×RIPA buffer (50 mM Tris-HCl, 150 mM NaCl, 1 mM EDTA, and 1% NP-40), supplemented with a complete mini protease inhibitor cocktail (Roche Applied Science, Indianapolis, IN, USA), and a phosphatase inhibitor cocktail (Sigma-Aldrich). Subsequently, total protein extracts were loaded and resolved on a 12% SDS-polyacrylamide gel, then transferred onto a polyvinyl difluoride membrane. After blocking with 5% skim milk in PBS/Tween 20 (0.1%) overnight at 4°C with specified primary antibodies, the membrane underwent PBS/Tween washes. Afterwards, the membrane was incubated with HRP-conjugated secondary antibodies for 1 h at 25°C and visualized using WestGlow™ PICO PLUS or FEMTO (Biomax Co., Ltd., Seoul, Korea).

### Statistical analysis

Statistical analyses were performed using Prism software (version 9.0; GraphPad Software, La Jolla, CA, USA). Dunnett’s multiple range test or Student’s t-tests were used to compare differences between two groups, whereas comparisons between multiple groups utilized the two-way ANOVA. All graphs are presented as mean ± standard error. A significance threshold of *p <*0.05 was set for all analyses.

## Results

### Aberrant expression of ABH antigen was observed in the skin of patients with AD

**Figure 1:**
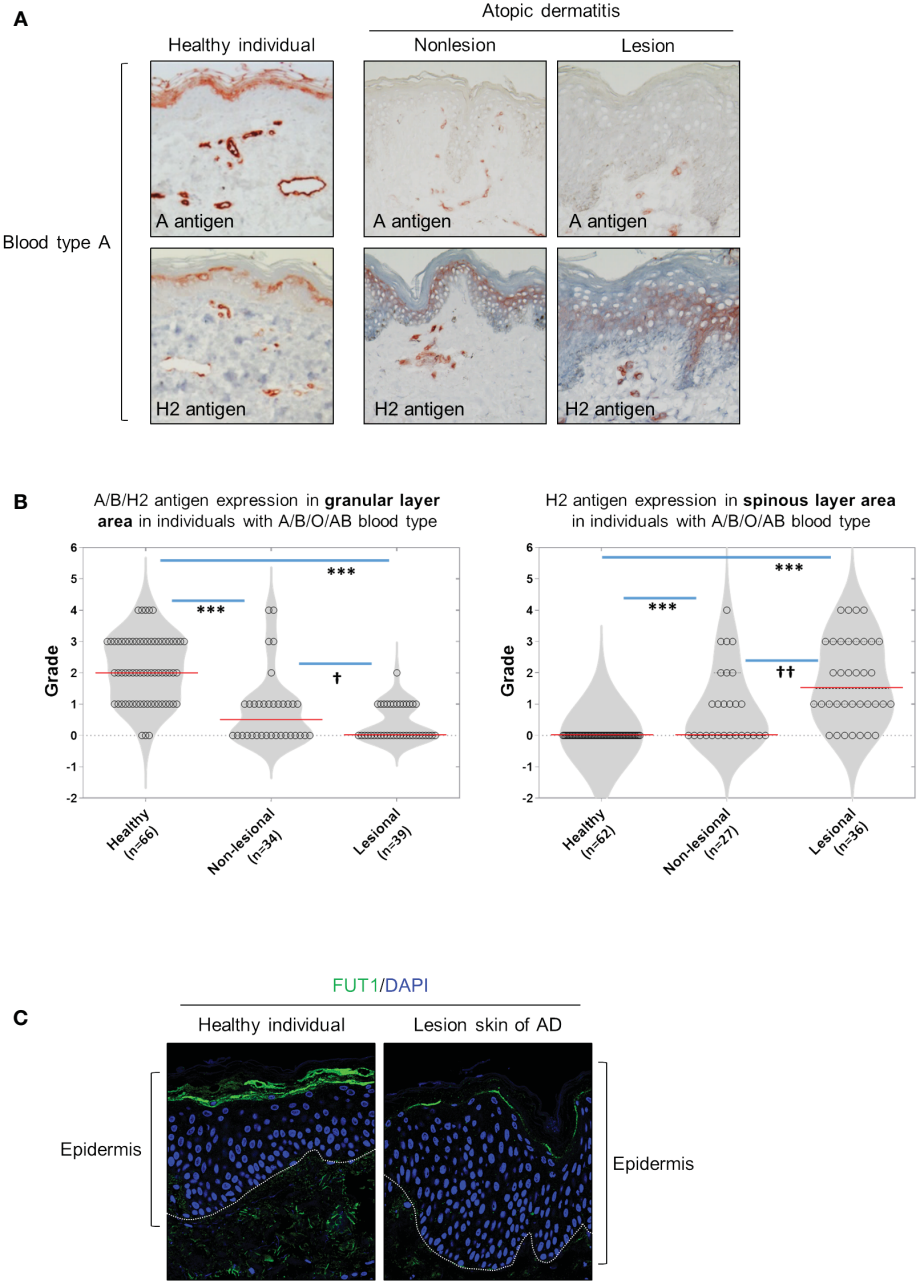
The granular layers of healthy skin exhibit ABH antigen and FUT1 expression which is diminished in nonlesional and lesional AD skin. **(A)** Immunohistochemical staining to detect A and H2 antigens in healthy skin, as well as on nonlesion and lesioned AD skin from individuals with blood type A. **(B)** Evaluating the expression of A, B, and H2 antigens in the granular (the left graph) and spinous layers (the right graph) of healthy skin, and comparing these expressions in nonlesion and lesional AD skin among individuals with blood types A, B, O, or AB. **(C)** Immunofluorescent labeling of FUT1 (green) and DAPI (blue) in healthy skin and lesioned AD skin. The data are representative of the mean ± SEM from 39 patients and 66 healthy controls. † < 0.05, †† p < 0.01, *** p < 0.001. AD, atopic dermatitis.

We have determined that ABH antigen expression was primarily localized in the granular and horny layers for each blood type (A, B, or O) of healthy skin (**Figure 1A**, **Supplementary Figure S1**). However, the expression of ABH antigens was absent or diminished in both the granular and horny layers of nonlesioned and lesioned skin from patients with AD (**Figure 1A**; **Supplementary Figure S1**). Moreover, H2 antigen expression abnormally manifested in the spinous layers, exhibiting a randomly dispersed pattern in many cases of patients with AD (**Figure 1A**; **Supplementary Figure S1**). The grading of immunostaining intensity revealed a significant decrease in the staining of A, B, and H2 antigens in granular layers in both lesional and nonlesional skin of patients with AD compared with those in healthy skin (**Figure 1B**). Additionally, the staining of H2 antigen in the spinous layers significantly increased in both lesional and nonlesional skin of patients with AD compared with that in healthy skin (**Figure 1B**). Notably, these changes were more pronounced in lesional skin than in nonlesional skin (**Figure 1B**). Furthermore, FUT1 expression was identified in the granular layers of healthy skin, while in the lesional skin of AD, it was either absent or reduced in these layers. Conversely, FUT1 expression exhibited no increase in the spinous layers of the lesional skin of AD (**Figure 1C**). The data showed a correlation between the patterns of FUT1 expression and H2 antigen expression in the granular layers, while no such correlation was observed in the spinous layers. These findings indicate the potential relationship between ABH antigen expression and inflammation in AD.

### FUT1 deficiency enhances AD-like inflammation in a mouse model

**Figure 2:**
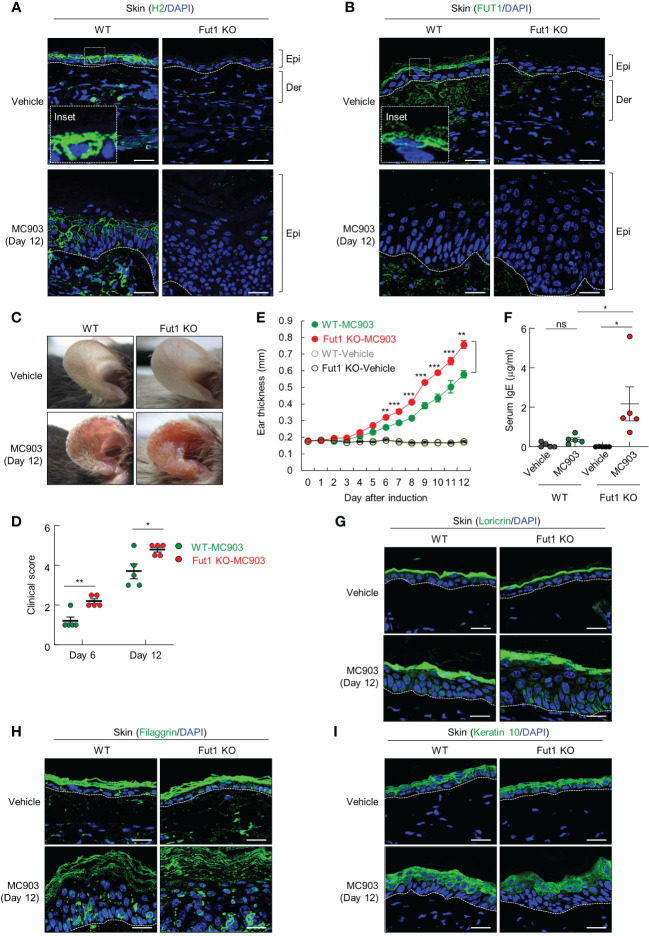
The deficiency of FUT1 exacerbates AD-like inflammation induced by MC903 in a murine model. **(A)** Immunofluorescent labeling of H2 antigen (green) and DAPI (blue) in the ears of both WT and *Fut1*-KO mice treated with either vehicle or MC903 on day 12. Scale bars, 20 μm. **(B)** Immunofluorescent labeling of FUT1 (green) and DAPI (blue) in ear sections from both WT and *Fut1*-KO mice. Scale bars, 20 μm. **(C)** Ear skin inflammation induced by MC903 in both WT and *Fut1*-KO mice on day 12. **(D)** Clinical scores of MC903-induced ear skin inflammation in both WT and *Fut1*-KO mice on days 6 and 12. **(E)** Measurement of ear swelling in both WT and *Fut1*-KO mice treated with MC903 using a digital caliper. **(F)** ELISA analysis of serum IgE levels in both WT and *Fut1*-KO mice treated with either vehicle or MC903 on day 12. **(G–I)** Immunofluorescent labeling of Loricrin, Filaggrin, or Keratin 10 (green), and DAPI (blue) in ear sections on day 12. The data are representative of the mean ± SEM of three independent experiments, with five mice per group. ns., not significant; * p < 0.05, ** p < 0.01, *** p < 0.001 and **** p < 0.0001. AD, atopic dermatitis; WT, wild-type.

Subsequently, we investigated the biological implications of ABH antigen expression in context of AD-like inflammation. To do this, we conducted a comparative analysis of MC903-induced AD-like inflammation in both WT and Fut1-deficient mice. H2 antigen and FUT1 expression were exhibited in the granular layers of control skin of WT mice, whereas they were notably absent in vehicle-treated Fut1-deficient mice (**Figures 2A, B**). The expression of the H2 antigen decreased in the granular layer, but increased in the spinous layers of WT mice treated with MC903 on day 12, whereas no such expression was observed in *Fut1*-knockout (KO) mice (**Figures 2A, B**). Additionally, the expression of Fut1 mRNA and protein in the skin of WT mice treated with MC903 decreased notably compared to the vehicle-treated skin of WT mice (**Supplementary Figures S2A, B**). Conversely, no expression of Fut1 mRNA and protein was detected in the skin of *Fut1*-KO mice treated with either vehicle or MC903 (**Supplementary Figures S2A, B**).

Upon daily application of 1 nmol MC903 or EtOH (vehicle) on the ears for 12 d, the ear skin of Fut1-deficient mice exhibited greater redness and attained higher clinical scores compared with that of WT mice (**Figures 2C, D**). From day 6 of MC903 application onward, the caliper measurements indicated a significantly higher ear thickness in Fut1-deficient mice compared with that of WT mice (**Figure 2E**). Fut1-deficient mice exhibited elevated serum IgE levels compared with that in WT mice following MC903 induction (**Figure 2F**). The expression levels of keratinocyte differentiation markers, such as Loricrin, Filaggrin, and Keratin 10, showed no significant differences between the epidermal layers of vehicle-treated WT and *Fut1-*KO mice, as well as between MC903-treated WT and *Fut1-*KO mice (**Figures 2G–I**). This indicates that the diminished expression of the H2 antigen was not caused by abnormal epidermal differentiation. Our findings suggest that FUT1 plays a crucial role in providing protection against AD-like inflammation.

### FUT1 deficiency exacerbates both epidermal and dermal thickening and inflammatory cell infiltration following MC903 application

**Figure 3:**
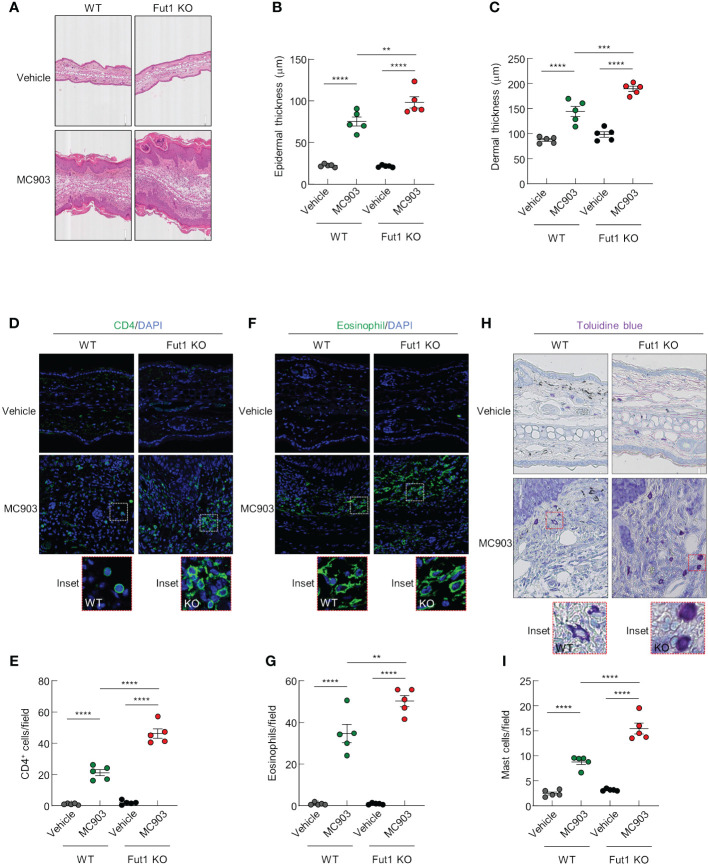
FUT1 deficiency amplifies MC903-induced ear thickening and immune cell infiltration. **(A)** H&E staining of the ear in both WT and *Fut1*-KO mice treated with either vehicle or MC903 on day 12. **(B)** Epidermal thicknesses measurements derived from H&E-stained ear sections. **(C)** Dermal thicknesses measurements obtained from H&E-stained ear sections. **(D)** Immunofluorescent labeling of CD4 (green) and DAPI (blue) in ear sections on day 12. **(E)** Quantitation of CD4^+^ T cells in immunofluorescent-labeled ear sections. **(F)** Immunofluorescent labeling of Eosinophil (green) and DAPI (blue) in ear sections on day 12. **(G)** Quantitation of eosinophils in immunofluorescent-labeled ear sections. **(H)** Toluidine blue staining of mast cells in ear sections. **(I)** Quantitation of mast cells in toluidine blue-stained ear sections. The data are representative of the mean ± SEM of three independent experiments, with five mice per group. ** p < 0.01, *** p < 0.001 and **** p < 0.0001. AD, atopic dermatitis; H&E, hematoxylin and eosin; WT, wild-type.

Histological analysis revealed increased inflammatory cell infiltration as well as greater epidermal and dermal thickening in Fut1-deficient mice compared with that in WT mice following MC903 application (**Figures 3A–C**). Nevertheless, in the control skin, there were no discernible differences in epidermal and dermal thickness between WT and Fut1-deficient mice (**Figures 3A–C**). To identify the specific immune cell types infiltrating the skin, we conducted staining on ear skin using CD4, Eosinophil marker, and toluidine blue for the detection of CD4^+^ T cells, eosinophils, and mast cells, respectively. The results revealed elevated numbers of infiltrating CD4^+^ T cells in the epidermis and dermis, along with increased counts of eosinophils and mast cells in the dermis of Fut1-deficient mice compared with those in WT mice following MC903 application (**Figures 3D–I**). However, no apparent differences in the numbers of CD4^+^ T cells, eosinophils, and mast cells were observed in the control skin between WT and Fut1-deficient mice (**Figures 3D–I**). Nevertheless, the expression levels of mast cell activation markers, F2RL1 and Mcpt6, exhibited variability depending on mast cell morphology in the MC903-treated skin of *Fut1*-KO mice (**Supplementary Figures S3A, B**). Our findings disclosed that Fut1 deficiency leads to heightened infiltration of inflammatory immune cells, concurrently resulting in increased dermal and epidermal thickening in AD-like inflammation.

### FUT1 deficiency amplifies the expression of AD-related cytokines and chemokines following MC903 application

**Figure 4:**
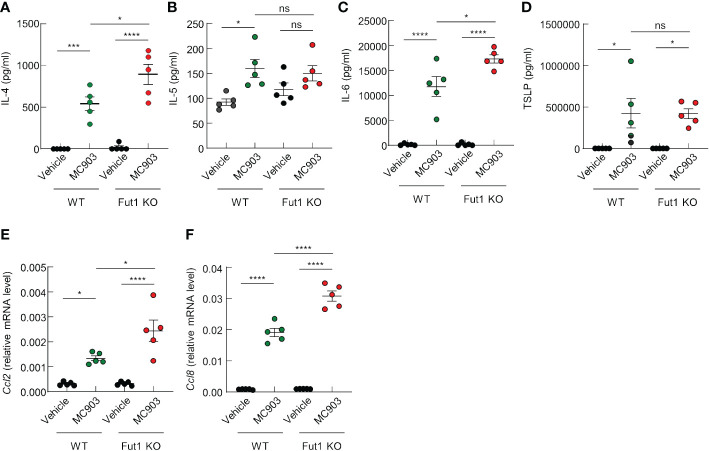
FUT1 deficiency enhances the production of AD-associated cytokines in ear skin induced by MC903. **(A–D)** ELISA analysis of IL-4 **(A)**, IL-5 **(B)**, IL-6 **(C)**, and TSLP **(D)** levels in the ear skin of both WT and *Fut1*-KO mice treated with either vehicle or MC903 on day 12. **(E, F)** Relative mRNA expression of *Ccl2* **(E)** and *Ccl8* **(F)** in the ear skin of both WT and *Fut1*-KO mice treated with vehicle or MC903 on day 12. The data are representative of the mean ± SEM of three independent experiments, with five mice per group. ns., not significant; * p < 0.05, *** p < 0.001 and **** p < 0.0001. AD, atopic dermatitis; TSLP, thymic stromal lymphopoietin.

We have discerned differences in the expression of AD-related cytokines and chemokines between WT and Fut1-deficient mice. ELISA results revealed elevated expression of AD-related cytokines, such as IL-4 and IL-6, in the skin of Fut1-deficient mice compared with that in WT mice following MC903 application (**Figures 4A, C**). Nevertheless, no noticeable differences were observed in the production of IL-5 and TSLP (**Figures 4B, D**). Quantitative PCR analysis showed a significant increase in the expression of *Ccl2* and *Ccl8* mRNA in the skin of Fut1-deficient mice compared with those in WT mice (**Figures 4E, F**). Our results conclusively demonstrated that the enhanced expression of chemoattractive factors, specifically CCL2 and CCL8, contributes to immune cell infiltration, subsequently leading to increased expression of IL-4 and IL-6 from immune cells in the skin of Fut1-deficient mice.

### FUT1 deficiency enhances the population of CD4^+^ T cells producing IL-4 in the ear-draining LNs following MC903 application

**Figure 5:**
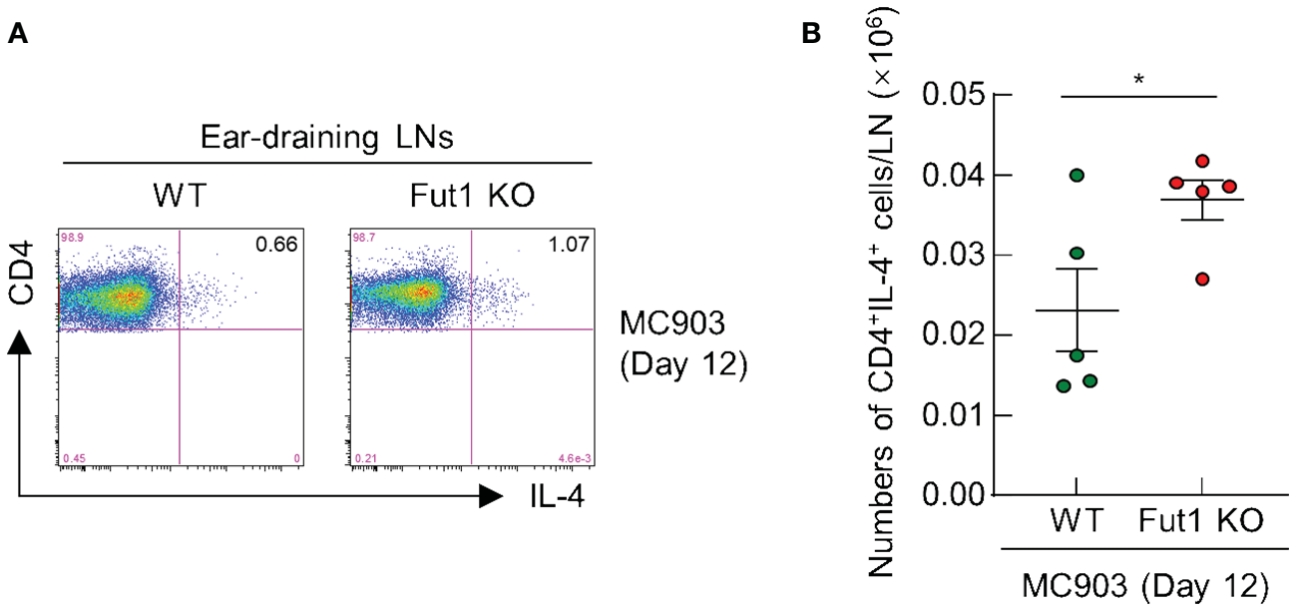
FUT1 deficiency heightens the number of IL-4-producing CD4^+^ T cells in the ear-draining LN following MC903 treatment. **(A)** FACS analysis of IL-4-producing CD4^+^ T cells in in the ear-draining LN after 12 d of MC903 treatment. **(B)** Quantification of IL-4-producing CD4^+^ T cells in the ear-draining LN on day 12. The data are representative of the mean ± SEM of two independent experiments, with five mice per group. * p < 0.05. LN, lymph node.

Finally, we performed FACS analysis to evaluate the population of IL-4 producing CD4^+^ T cells in the ear-draining LNs of both WT and *Fut1-*KO mice after a 12-d treatment with MC903. The results revealed an elevated ratio and greater numbers of IL-4 producing CD4^+^ T cells in the ear-draining LNs of *Fut1*-KO mice compared with those in WT mice (**Figures 5A, B**). This observation correlated with elevated levels of IL-4 production and a greater number of CD4^+^ T cells in the skin of *Fut1-*KO mice compared with that in WT mice. This suggests an increased proliferation of IL-4 producing CD4^+^ T cells in the ear-draining LNs.

## Discussion

In this study, we elucidated the protective role of H2 antigen expression, induced by FUT1, in the epidermal layers against AD-like inflammation triggered by MC903 treatment in a murine model. Additionally, we demonstrated the diminished expression of ABH antigens in the granular and horny layers of lesional skin in AD compared with that in healthy skin. However, there is an observed increase in H2 antigen expression in spinous layers of lesional skin in AD. This result aligns with our earlier findings, indicating a significant decrease in the expression of ABH antigens in the granular layers of photoaged skin and acutely UV-irradiated skin (33). Notably, there is an elevation in H2 antigen expression in the spinous layers of these skin conditions (33). Our findings suggest a robust link between skin inflammation and the diminished expression of ABH antigens in the epidermal granular layers, accompanied by an elevation of H2 antigens in the spinous layer. This heightened expression of the H2 antigen, a precursor to A and B antigens, in the spinous layers of inflamed skin may represent compensatory mechanisms in response to the reduced expression of ABH antigens in the granular and horny layers. Alternatively, it could be attributed to a decrease in ABO glycosylatransferase expression or activation. Consequently, this may result in the accumulation of H2 antigen without its conversion into A or B antigens. Previous investigations showed a decrease in *ABO* gene expression in ovarian tumor tissues and human bladder tumors (41, 45). The ABO polymorphism has been linked to specific infectious diseases, such as *Helicobacter pylori* infection, *Plasmodium falciparum* malaria, and SARS-CoV-2 (46–49). These findings underscore the broader clinical relevance of the ABH antigens, reaching beyond their role in blood transfusion and transplantation. Moreover, these antigens play a crucial role in various clinically significant aspects, including inflammatory responses, cancer, and other diseases (50–53).

Furthermore, our results suggest that the regulation of H2 antigen expression in the skin epidermal layers is predominantly governed by FUT1, as demonstrated using *Fut1*-KO mice. Fut1-deficient mice do not exhibit H2 antigen expression in the granular layers of control skin. FUT1 expression was also observed in the granular layers of healthy human and mouse skin. However, FUT1 expression was notably absent in the spinous layers of AD and MC903-treated mice skin, despite an elevated expression of the H2 antigen in these layers. This observation could be a potential short half-life for FUT1 expression in the spinous layers following H2 antigen induction. Further elucidation is needed to clarify this aspect. In addition, our confirmation that MC903 treatment results in decreased levels of Fut1 mRNA and protein in the skin suggests direct involvement of MC903 in downregulating the H2 antigen expression. This cumulative evidence suggests that the control of H2 expression in the skin is predominantly controlled by FUT1. Importantly, this downregulation does not result from abnormal epidermal differentiation, as evidenced by the expression of epidermal differentiation markers, including loricrin, filaggrin, and keratin 10, within the granular and horny layers of both vehicle and MC903-treated WT and *Fut1-*KO mice. This implies that the absence of H2 antigen expression in the granular and horny layers of MC903-treated skin does not stem from impaired terminal differentiation after MC903 treatment.

Under physiological conditions, the H1 antigen in the intestine serves a protective role by maintaining a balance of commensal bacteria (54, 55). Loss-of-function mutations in FUT2 result in an altered gut microbiome, contributing to the development of inflammatory bowel diseases (55, 56). Nevertheless, amid the inflammatory response, the function of the H1 antigen in the intestine and lung remains controversial, suggesting its potential involvement in either mitigating or exacerbating inflammation (57–62). Our findings indicate that H2 antigens contribute to a protective role against AD-like inflammation triggered by MC903 in the skin. In the absence of H2 antigens in *Fut1*-KO mice, MC903-induced ear swelling, as well as epidermal and dermal thickening, exceeded that observed in WT mice. Corresponding with the heightened dermal thickening, the infiltration of immune cells, including CD4^+^ T cells, eosinophils, and mast cells into the dermis, was more pronounced in *Fut1*-KO mice compared with those in WT mice.

The topical application of MC903 on the skin swiftly initiates TSLP production, considered as an initial trigger for Th2 inflammation, in keratinocytes, beginning on day 2 after the initial application (44). Nevertheless, our study revealed no apparent distinction in TSLP production between the skin of WT and *Fut1*-KO mice on day 12, a relatively late time point. This observation could be attributed to the substantial accumulation of TSLP production over the 12-day period with daily application of MC903. IL-4 is primarily produced by Th2 cells and, as non-T cells, by eosinophils (63, 64). The production of IL-4, occurring in the late response following MC903 application, was more pronounced in *Fut1*-KO mice compared with that in WT mice. This correlated with a higher number of infiltrating CD4^+^ T cells and eosinophils in the skin of *Fut1*-KO mice compared with those in WT mice. IL-4 stimulates the expansion and activation of mast cells, as well as induces B cells to produce IgE (65–70). This aligns with our findings, which demonstrate higher IgE production and a greater number of mast cells in *Fut1-*KO mice compared with that in WT mice. A study has elucidated that mast cells in MC903-induced AD-like skin are sustained through both the local proliferation of tissue-resident mast cells and the infiltration/differentiation of bone marrow-derived mast cell progenitors. This finding provides a novel perspective on mast cell heterogeneity in allergic conditions (71). Such heterogeneity may contribute to the variability observed in mast cell morphology and the different expression levels of mast cell activation markers (F2RL1 and Mcpt6) in MC903-treated skin in our study, despite the ongoing ambiguity surrounding the functional distinctions between these cell types.

A prior study demonstrated elevated IL-6 production by T cells derived from patients with AD (72). Additionally, IL-6 is generated by various cell types, including dendritic cells, mast cells, eosinophils, and keratinocytes (73–76). After the application of MC903 in *Fut1-*KO mice, there was a significantly elevated production of IL-6 in the skin, along with increased infiltration of mast cells and eosinophils compared to WT mice. IL-5 is recognized as the most crucial cytokine in eosinophil maturation within the bone marrow and their subsequent release into the blood (77–79). In humans, IL-5 affects eosinophils and basophils, influencing their growth, activation, and survival (80, 81). However, our findings indicate that IL-5 production did not exhibit a significant difference between the skin of WT and *Fut1*-KO mice. This implies that in mice, alternative chemokines may exert a substantial influence on eosinophil recruitment into the skin, or it could be essential to explore different time points to discern any divergence in IL-5 production. Furthermore, our findings indicate that the expression of chemokines, CCL2 and CCL8, was notably higher in the skin of *Fut1*-KO mice compared with those in WT mice on day 12 after MC903 application. CCL8 has the potential to regulate the recruitment of immune cells in various tissues during inflammatory diseases (82). Mouse CCL8 is constitutively expressed in the skin and may contribute to chronic eosinophilic inflammation by inducing the accumulation of CD4^+^ Th2 cells in a mouse model of chronic AD (24). CCL2 secretion in the skin plays a role in recruiting inflammatory monocytes, dendritic cells, and memory T cells (22, 83–85). Mice deficient in CCL2 exhibited diminished recruitment of monocytes and eosinophils in an irritant contact dermatitis model (86). CCL2 induces DC maturation and migration from the skin to draining lymph nodes, facilitating antigen presentation to T cells (87). The data strongly support our findings, indicating that heightened production of CCL2 and CCL8 is likely to contribute to the augmented recruitment of immune cells into the skin of *Fut1*-KO mice. Additionally, our results reveal an increased presence of IL-4-producing CD4^+^ T cells in the ear-draining LNs of *Fut1*-KO mice compared with that in WT mice.

We investigated the synthesis of the H2 antigen by Fut1 and its expression patterns within healthy human and murine skin, particularly within the granular cells and stratum corneum. Notably, these patterns differed in skin lesions in patients with AD and in MC903-treated mice. In these pathological contexts, both H2 antigen levels and FUT1 expression within the granular layers and stratum corneum were markedly decreased but were significantly increased in the spinous layers of lesional skin. Based on these findings, complete loss of H2 antigen expression in patients with AD may exacerbate inflammation associated with this condition. Although inflammation typically correlates with reduced H2 antigen expression in the granular cells and stratum corneum of healthy skin, compensatory upregulation of H2 antigen expression throughout the spinous layers in lesional skin suggests a mechanism for counteracting this loss. Further studies are required to fully understand the underlying mechanisms.

The importance of Fut1 or the H2 antigen in biological processes may vary depending on the context. Both Fut1-mediated terminal fucosylation and the resultant H2 antigen may contribute distinct but interconnected roles in cellular interactions and signaling pathways. The presence or absence of FUT1 can influence interactions between cells and molecules in the immune system, as well as cell adhesion and signaling (88–91). The absence of functional FUT1 enzyme activity leads to the Bombay phenotype, in which individuals lack H2 antigen expression; this absence can have important biological consequences (92, 93). Similarly, the presence of the H2 antigen and its modifications may contribute to various physiological processes (94, 95).

In summary, our study reveals that ABH antigens are expressed in the granular and honey layers of healthy human and mice skin. However, in the context of AD and MC903-treated mice skin, there is a significant reduction in the expression of these antigens within these layers. Notably, our findings indicate that the H2 antigen plays a role in a protective capacity against AD-like inflammation, leading to reductions in clinical scores, skin thickening, and the production of AD-related cytokines. While our results underscore the potential therapeutic significance of the H2 antigen in AD by regulating blood group antigens, further investigation is essential to elucidate the specific mechanisms through which the H2 antigen contributes to its protective role in AD inflammation.

## Data availability statement

The original contributions presented in the study are included in the article/**Supplementary Material**. Further inquiries can be directed to the corresponding authors.

## Ethics statement

The studies involving humans were approved by The Institutional Review Board (IRB No. C-1312-084-543) of Seoul National University Hospital. The studies were conducted in accordance with the local legislation and institutional requirements. The participants provided their written informed consent to participate in this study. The animal study was approved by The Animal Care and Use Committee (IACUC No. 20-0112-S1A0) of Seoul National University Hospital. The study was conducted in accordance with the local legislation and institutional requirements.

## Author contributions

YL: Conceptualization, Data curation, Investigation, Methodology, Writing – original draft, Writing – review & editing. NL: Data curation, Investigation, Writing – review & editing. J-HO: Writing – review & editing, Funding acquisition, Investigation. JS: Resources, Writing – review & editing. S-PJ: Data curation, Writing – review & editing. DL: Conceptualization, Funding acquisition, Writing – review & editing. JC: Conceptualization, Funding acquisition, Supervision, Writing – review & editing.

## Supplementary material

Supplementary Figure 1 The granular layers of healthy skin exhibit ABH antigen expression which is diminished in nonlesional and lesional AD skin. Immunohistochemical staining to detect B and H antigens in healthy skin, as well as on nonlesion and lesioned AD skin from individuals with blood types B or O. AD, atopic dermatitis.

Supplementary Figure 2 Reduced expression levels of Fut1 mRNA and protein in the skin of WT mice treated with MC903. **(A)** Relative mRNA expression of *Fut1* in the ear skin of both WT and *Fut1*-KO mice treated with vehicle or MC903 on day 12. **(B)** The protein expression of Fut1 and β-Actin in the ear skin of both WT and *Fut1*-KO mice treated with vehicle or MC903 on day 12. The data represent the mean ± SEM of three independent experiments, with five mice per group. ns., not significant; **** p < 0.0001.

Supplementary Figure 3 Expression of mast cell activation markers in the skin. **(A)** Immunofluorescent labeling of F2RL1 (green) and DAPI (blue) in ear sections on day 12. **(B)** Immunofluorescent labeling of Mcpt6 (green) and DAPI (blue) in ear sections on day 12.

## References

[1] Langan SM Irvine AD Weidinger S. Atopic dermatitis. Lancet. (2020) 396:345–60. doi: 10.1016/S0140-6736(20)31286-1 32738956

[2] Odhiambo JA Williams HC Clayton TO Robertson CF Asher MI Group IPTS. Global variations in prevalence of eczema symptoms in children from ISAAC Phase Three. J Allergy Clin Immunol. (2009) 124:1251–8 e23. doi: 10.1016/j.jaci.2009.10.009 20004783

[3] Silverberg JI Hanifin JM. Adult eczema prevalence and associations with asthma and other health and demographic factors: a US population-based study. J Allergy Clin Immunol. (2013) 132:1132–8. doi: 10.1016/j.jaci.2013.08.031 24094544

[4] Angles MV Antonietti CA Torre AC Juszkiewicz Franze E Mazzuoccolo LD Parisi CAS. Prevalence of atopic dermatitis in adults. Bras Dermatol. (2022) 97:107–9. doi: 10.1016/j.abd.2020.10.016 PMC8799860 34839985

[5] Bieber T. Atopic dermatitis: an expanding therapeutic pipeline for a complex disease. Nat Rev Drug Discov. (2022) 21:21–40. doi: 10.1038/s41573-021-00266-6 34417579 PMC8377708

[6] Kapur S Watson W Carr S. Atopic dermatitis. Allergy Asthma Clin Immunol. (2018) 14:52. doi: 10.1186/s13223-018-0281-6 30275844 PMC6157251

[7] Egawa G Kabashima K. Multifactorial skin barrier deficiency and atopic dermatitis: Essential topics to prevent the atopic march. J Allergy Clin Immunol. (2016) 138:350–358 e1. doi: 10.1016/j.jaci.2016.06.002 27497277

[8] Nomura T Kabashima K. Advances in atopic dermatitis in 2015. J Allergy Clin Immunol. (2016) 138:1548–55. doi: 10.1016/j.jaci.2016.10.004 27931536

[9] Fonacier LS Dreskin SC Leung DY. Allergic skin diseases. J Allergy Clin Immunol. (2010) 125:S138–49. doi: 10.1016/j.jaci.2009.05.039 19932921

[10] Mansouri Y Guttman-Yassky E. Immune pathways in atopic dermatitis, and definition of biomarkers through broad and targeted therapeutics. J Clin Med. (2015) 4:858–73. doi: 10.3390/jcm4050858 PMC4470203 26239452

[11] Moreno AS McPhee R Arruda LK Howell MD. Targeting the T helper 2 inflammatory axis in atopic dermatitis. Int Arch Allergy Immunol. (2016) 171:71–80. doi: 10.1159/000451083 27846627

[12] Gittler JK Shemer A Suarez-Farinas M Fuentes-Duculan J Gulewicz KJ Wang CQ. Progressive activation of T(H)2/T(H)22 cytokines and selective epidermal proteins characterizes acute and chronic atopic dermatitis. J Allergy Clin Immunol. (2012) 130:1344–54. doi: 10.1016/j.jaci.2012.07.012 PMC3991245 22951056

[13] Lebman DA Coffman RL. Interleukin 4 causes isotype switching to IgE in T cell-stimulated clonal B cell cultures. J Exp Med. (1988) 168:853–62. doi: 10.1084/jem.168.3.853 PMC2189023 3049907

[14] Junttila IS. Tuning the cytokine responses: an update on interleukin (IL)-4 and IL-13 receptor complexes. Front Immunol. (2018) 9:888. doi: 10.3389/fimmu.2018.00888 29930549 PMC6001902

[15] Leiferman KM. Eosinophils in atopic dermatitis. J Allergy Clin Immunol. (1994) 94:1310–7. doi: 10.1016/0091-6749(94)90347-6 7798571

[16] Kawakami T Ando T Kimura M Wilson BS Kawakami Y. Mast cells in atopic dermatitis. Curr Opin Immunol. (2009) 21:666–78. doi: 10.1016/j.coi.2009.09.006 PMC2839879 19828304

[17] Keith YH Egawa G Honda T Kabashima K. Mast cells in type 2 skin inflammation: Maintenance and function. Eur J Immunol. (2023) 53:e2250359. doi: 10.1002/eji.202250359 36933268

[18] Kostenis E Ulven T. Emerging roles of DP and CRTH2 in allergic inflammation. Trends Mol Med. (2006) 12:148–58. doi: 10.1016/j.molmed.2006.02.005 16545607

[19] Liu FT Goodarzi H Chen HY. IgE, mast cells, and eosinophils in atopic dermatitis. Clin Rev Allergy Immunol. (2011) 41:298–310. doi: 10.1007/s12016-011-8252-4 21249468

[20] Vestergaard C Just H Baumgartner Nielsen J Thestrup-Pedersen K Deleuran M. Expression of CCR2 on monocytes and macrophages in chronically inflamed skin in atopic dermatitis and psoriasis. Acta Derm Venereol. (2004) 84:353–8. doi: 10.1080/00015550410034444 15370700

[21] Rebane A Zimmermann M Aab A Baurecht H Koreck A Karelson M. Mechanisms of IFN-gamma-induced apoptosis of human skin keratinocytes in patients with atopic dermatitis. J Allergy Clin Immunol. (2012) 129:1297–306. doi: 10.1016/j.jaci.2012.02.020 22445417

[22] Novoszel P Holcmann M Stulnig G De Sa Fernandes C Zyulina V Borek I. Psoriatic skin inflammation is promoted by c-Jun/AP-1-dependent CCL2 and IL-23 expression in dendritic cells. EMBO Mol Med. (2021) 13:e12409. doi: 10.15252/emmm.202012409 33724710 PMC8033525

[23] Jiang H Cui H Wang T Shimada SG Sun R Tan Z. CCL2/CCR2 signaling elicits itch- and pain-like behavior in a murine model of allergic contact dermatitis. Brain Behav Immun. (2019) 80:464–73. doi: 10.1016/j.bbi.2019.04.026 30981714

[24] Islam SA Chang DS Colvin RA Byrne MH McCully ML Moser B. Mouse CCL8, a CCR8 agonist, promotes atopic dermatitis by recruiting IL-5+ T(H)2 cells. Nat Immunol. (2011) 12:167–77. doi: 10.1038/ni.1984 PMC3863381 21217759

[25] Guzik TJ Bzowska M Kasprowicz A Czerniawska-Mysik G Wojcik K Szmyd D. Persistent skin colonization with Staphylococcus aureus in atopic dermatitis: relationship to clinical and immunological parameters. Clin Exp Allergy. (2005) 35:448–55. doi: 10.1111/j.1365-2222.2005.02210.x 15836752

[26] Tauber M Balica S Hsu CY Jean-Decoster C Lauze C Redoules D. Staphylococcus aureus density on lesional and nonlesional skin is strongly associated with disease severity in atopic dermatitis. J Allergy Clin Immunol. (2016) 137:1272–1274 e3. doi: 10.1016/j.jaci.2015.07.052 26559326

[27] Reche PA Soumelis V Gorman DM Clifford T Liu M Travis M. Human thymic stromal lymphopoietin preferentially stimulates myeloid cells. J Immunol. (2001) 167:336–43. doi: 10.4049/jimmunol.167.1.336 11418668

[28] Soumelis V Reche PA Kanzler H Yuan W Edward G Homey B. Human epithelial cells trigger dendritic cell mediated allergic inflammation by producing TSLP. Nat Immunol. (2002) 3:673–80. doi: 10.1038/ni805 12055625

[29] He R Oyoshi MK Garibyan L Kumar L Ziegler SF Geha RS. TSLP acts on infiltrating effector T cells to drive allergic skin inflammation. Proc Natl Acad Sci USA. (2008) 105:11875–80. doi: 10.1073/pnas.0801532105 PMC2575291 18711124

[30] Apoil PA Roubinet F Despiau S Mollicone R Oriol R Blancher A. Evolution of alpha 2-fucosyltransferase genes in primates: relation between an intronic Alu-Y element and red cell expression of ABH antigens. Mol Biol Evol. (2000) 17:337–51. doi: 10.1093/oxfordjournals.molbev.a026314 10723735

[31] Costache M Apoil PA Cailleau A Elmgren A Larson G Henry S. Evolution of fucosyltransferase genes in vertebrates. J Biol Chem. (1997) 272:29721–8. doi: 10.1074/jbc.272.47.29721 9368041

[32] Oriol R Le Pendu J Mollicone R. Genetics of ABO, H, Lewis, X and related antigens. Vox Sang. (1986) 51:161–71. doi: 10.1159/000461485 2433836

[33] Lee DH Jung JY Oh JH Lee S Kim YK Chung JH. Ultraviolet irradiation modulates ABO blood group antigens in human skin in vivo: possible implication in skin aging. J Dermatol Sci. (2012) 66:71–3. doi: 10.1016/j.jdermsci.2012.01.006 22386697

[34] Ravn V Dabelsteen E. Tissue distribution of histo-blood group antigens. APMIS. (2000) 108:1–28. doi: 10.1034/j.1600-0463.2000.d01-1.x 10698081

[35] Henry SM. Molecular diversity in the biosynthesis of GI tract glycoconjugates. A blood-group-related chart of microorganism receptors. Transfus Clin Biol. (2001) 8:226–30. doi: 10.1016/S1246-7820(01)00112-4 11499965

[36] Wang X Zhang F Jiang Y Xu Z Feng X Li L. Highly individual- and tissue-specific expression of glycoprotein group A and B blood antigens in the human kidney and liver. BMC Immunol. (2021) 22:66. doi: 10.1186/s12865-021-00456-2 34598667 PMC8485463

[37] Finne J Breimer ME Hansson GC Karlsson KA Leffler H Vliegenthart JF. Novel polyfucosylated N-linked glycopeptides with blood group A, H, X, and Y determinants from human small intestinal epithelial cells. J Biol Chem. (1989) 264:5720–35. doi: 10.1016/S0021-9258(18)83609-2 2466830

[38] Bjork S Breimer ME Hansson GC Karlsson KA Leffler H. Structures of blood group glycosphingolipids of human small intestine. A relation between the expression of fucolipids of epithelial cells and the ABO, Le and Se phenotype of the donor. J Biol Chem. (1987) 262:6758–65. doi: 10.1016/S0021-9258(18)48309-3 3571286

[39] Stowell CP Stowell SR. Biologic roles of the ABH and Lewis histo-blood group antigens Part I: infection and immunity. Vox Sang. (2019) 114:426–42. doi: 10.1111/vox.12787 31070258

[40] Zouine S Marnissi F Otmani N Bennani Othmani M Zaid N Kojok K. Expression of histo-blood group antigens in tumor and adjacent normal breast tissues as prognostic markers of breast carcinoma. J Breast Cancer. (2020) 23:69–79. doi: 10.4048/jbc.2020.23.e13 32140271 PMC7043947

[41] Wang C Zhou J Wang L Xing T Dai H Zhou Y. ABO blood groups and expression of blood group antigens of epithelial ovarian cancer in Chinese women. Cancer Med. (2023) 12:7498–507. doi: 10.1002/cam4.5476 PMC10067109 36415180

[42] Rizzo AN Schmidt EP. ABO blood type: a window into the genetics of acute respiratory distress syndrome susceptibility. J Clin Invest. (2021) 131: e144075. doi: 10.1172/JCI144075 33141764 PMC7773403

[43] Cheng Y Cheng G Chui CH Lau FY Chan PK Ng MH. ABO blood group and susceptibility to severe acute respiratory syndrome. JAMA. (2005) 293:1450–1. doi: 10.1001/jama.293.12.1450-c 15784866

[44] Li M Hener P Zhang Z Kato S Metzger D Chambon P. Topical vitamin D3 and low-calcemic analogs induce thymic stromal lymphopoietin in mouse keratinocytes and trigger an atopic dermatitis. Proc Natl Acad Sci USA. (2006) 103:11736–41. doi: 10.1073/pnas.0604575103 PMC1544239 16880407

[45] Orntoft TF Meldgaard P Pedersen B Wolf H. The blood group ABO gene transcript is down-regulated in human bladder tumors and growth-stimulated urothelial cell lines. Cancer Res. (1996) 56:1031–36. 8640757

[46] Boren T Falk P Roth KA Larson G Normark S. Attachment of Helicobacter pylori to human gastric epithelium mediated by blood group antigens. Science. (1993) 262:1892–5. doi: 10.1126/science.8018146 8018146

[47] Cserti CM Dzik WH. The ABO blood group system and Plasmodium falciparum malaria. Blood. (2007) 110:2250–8. doi: 10.1182/blood-2007-03-077602 17502454

[48] Abegaz SB. Human ABO blood groups and their associations with different diseases. BioMed Res Int. (2021) 2021:6629060. doi: 10.1155/2021/6629060 33564677 PMC7850852

[49] Butler EA Parikh R Grandi SM Ray JG Cohen E. ABO and Rh blood groups and risk of infection: systematic review and meta-analysis. BMC Infect Dis. (2023) 23:797. doi: 10.1186/s12879-023-08792-x 37964217 PMC10647048

[50] Isozaki T Amin MA Ruth JH Campbell PL Tsou PS Ha CM. Fucosyltransferase 1 mediates angiogenesis in rheumatoid arthritis. Arthritis Rheumatol. (2014) 66:2047–58. doi: 10.1002/art.38648 PMC4426876 24692243

[51] Kim KW Ryu JS Ko JH Kim JY Kim HJ Lee HJ. FUT1 deficiency elicits immune dysregulation and corneal opacity in steady state and under stress. Cell Death Dis. (2020) 11:285. doi: 10.1038/s41419-020-2489-x 32332708 PMC7181665

[52] Jung JY Oh JH Lee DH Lee S Chung JH. Blood type B antigen modulates cell migration through regulating cdc42 expression and activity in HaCaT cells. J Cell Physiol. (2013) 228:2243–51. doi: 10.1002/jcp.24393 23625752

[53] Li N Lee Y Suh JH Oh JH Jin SP Lee DH. Fucosylation deficiency enhances imiquimod-induced psoriasis-like skin inflammation by promoting CXCL1 expression. Biochim Biophys Acta Mol Basis Dis. (2024) 1870:166988. doi: 10.1016/j.bbadis.2023.166988 38070583

[54] Goto Y Obata T Kunisawa J Sato S Ivanov II Lamichhane A. Innate lymphoid cells regulate intestinal epithelial cell glycosylation. Science. (2014) 345:1254009. doi: 10.1126/science.1254009 25214634 PMC4774895

[55] Cheng S Hu J Wu X Pan JA Jiao N Li Y. Altered gut microbiome in FUT2 loss-of-function mutants in support of personalized medicine for inflammatory bowel diseases. J Genet Genomics. (2021) 48:771–80. doi: 10.1016/j.jgg.2021.08.003 34419617

[56] Tong M McHardy I Ruegger P Goudarzi M Kashyap PC Haritunians T. Reprograming of gut microbiome energy metabolism by the FUT2 Crohn's disease risk polymorphism. ISME J. (2014) 8:2193–206. doi: 10.1038/ismej.2014.64 PMC4992076 24781901

[57] Innes AL McGrath KW Dougherty RH McCulloch CE Woodruff PG Seibold MA. The H antigen at epithelial surfaces is associated with susceptibility to asthma exacerbation. Am J Respir Crit Care Med. (2011) 183:189–94. doi: 10.1164/rccm.201003-0488OC PMC3040389 20732988

[58] Saku A Hirose K Ito T Iwata A Sato T Kaji H. Fucosyltransferase 2 induces lung epithelial fucosylation and exacerbates house dust mite-induced airway inflammation. J Allergy Clin Immunol. (2019) 144:698–709 e9. doi: 10.1016/j.jaci.2019.05.010 31125592

[59] Le Pendu J Ruvoen-Clouet N Kindberg E Svensson L. Mendelian resistance to human norovirus infections. Semin Immunol. (2006) 18:375–86. doi: 10.1016/j.smim.2006.07.009 PMC7129677 16973373

[60] Thorven M Grahn A Hedlund KO Johansson H Wahlfrid C Larson G. A homozygous nonsense mutation (428G–>A) in the human secretor (FUT2) gene provides resistance to symptomatic norovirus (GGII) infections. J Virol. (2005) 79:15351–5. doi: 10.1128/JVI.79.24.15351-15355.2005 PMC1315998 16306606

[61] Carlsson B Kindberg E Buesa J Rydell GE Lidon MF Montava R. The G428A nonsense mutation in FUT2 provides strong but not absolute protection against symptomatic GII.4 Norovirus infection. PloS One. (2009) 4:e5593. doi: 10.1371/journal.pone.0005593 19440360 PMC2680586

[62] Blackwell CC Jonsdottir K Hanson MF Weir DM. Non-secretion of ABO blood group antigens predisposing to infection by Haemophilus influenzae. Lancet. (1986) 2:687. doi: 10.1016/S0140-6736(86)90193-5 2876155

[63] Nonaka M Nonaka R Woolley K Adelroth E Miura K Okhawara Y. Distinct immunohistochemical localization of IL-4 in human inflamed airway tissues. IL-4 is localized to eosinophils in vivo and is released by peripheral blood eosinophils. J Immunol. (1995) 155:3234–44. doi: 10.4049/jimmunol.155.6.3234 7673736

[64] Kolbinger A Schaufele TJ Steigerwald H Friedel J Pierre S Geisslinger G. Eosinophil-derived IL-4 is necessary to establish the inflammatory structure in innate inflammation. EMBO Mol Med. (2023) 15:e16796. doi: 10.15252/emmm.202216796 36541656 PMC9906331

[65] Burton OT Darling AR Zhou JS Noval-Rivas M Jones TG Gurish MF. Direct effects of IL-4 on mast cells drive their intestinal expansion and increase susceptibility to anaphylaxis in a murine model of food allergy. Mucosal Immunol. (2013) 6:740–50. doi: 10.1038/mi.2012.112 PMC3600405 23149659

[66] Nabeshima Y Hiragun T Morita E Mihara S Kameyoshi Y Hide M. IL-4 modulates the histamine content of mast cells in a mast cell/fibroblast co-culture through a Stat6 signaling pathway in fibroblasts. FEBS Lett. (2005) 579:6653–8. doi: 10.1016/j.febslet.2005.09.104 16298365

[67] Granato A Hayashi EA Baptista BJ Bellio M Nobrega A. IL-4 regulates Bim expression and promotes B cell maturation in synergy with BAFF conferring resistance to cell death at negative selection checkpoints. J Immunol. (2014) 192:5761–75. doi: 10.4049/jimmunol.1300749 24835393

[68] Thyphronitis G Tsokos GC June CH Levine AD Finkelman FD. IgE secretion by Epstein-Barr virus-infected purified human B lymphocytes is stimulated by interleukin 4 and suppressed by interferon gamma. Proc Natl Acad Sci USA. (1989) 86:5580–4. doi: 10.1073/pnas.86.14.5580 PMC297666 2546158

[69] Gauchat JF Lebman DA Coffman RL Gascan H de Vries JE. Structure and expression of germline epsilon transcripts in human B cells induced by interleukin 4 to switch to IgE production. J Exp Med. (1990) 172:463–73. doi: 10.1084/jem.172.2.463 PMC2188335 1695667

[70] Wu CY Sarfati M Heusser C Fournier S Rubio-Trujillo M Peleman R. Glucocorticoids increase the synthesis of immunoglobulin E by interleukin 4-stimulated human lymphocytes. J Clin Invest. (1991) 87:870–7. doi: 10.1172/JCI115092 PMC329876 1825666

[71] Keith YH Egawa G Honda T Kabashima K. Infiltration and local differentiation of bone marrow-derived integrinbeta7-positive mast cell progenitors in atopic dermatitis-like skin. J Allergy Clin Immunol. (2023) 151:159–171 e8. doi: 10.1016/j.jaci.2022.09.011 36122789

[72] Toshitani A Ansel JC Chan SC Li SH Hanifin JM. Increased interleukin 6 production by T cells derived from patients with atopic dermatitis. J Invest Dermatol. (1993) 100:299–304. doi: 10.1111/1523-1747.ep12469875 8440909

[73] Ilves T Harvima IT. Decrease in chymase activity is associated with increase in IL-6 expression in mast cells in atopic dermatitis. Acta Derm Venereol. (2015) 95:411–6. doi: 10.2340/00015555-1979 25270893

[74] Lacy P Levi-Schaffer F Mahmudi-Azer S Bablitz B Hagen SC Velazquez J. Intracellular localization of interleukin-6 in eosinophils from atopic asthmatics and effects of interferon gamma. Blood. (1998) 91:2508–16. doi: 10.1182/blood.V91.7.2508.2508_2508_2516 9516152

[75] Oh S Chung H Chang S Lee SH Seok SH Lee H. Effect of mechanical stretch on the DNCB-induced proinflammatory cytokine secretion in human keratinocytes. Sci Rep. (2019) 9:5156. doi: 10.1038/s41598-019-41480-y 30914685 PMC6435715

[76] Yao W Chang J Sehra S Travers JB Chang CH Tepper RS. Altered cytokine production by dendritic cells from infants with atopic dermatitis. Clin Immunol. (2010) 137:406–14. doi: 10.1016/j.clim.2010.09.001 PMC2975770 20880754

[77] Clutterbuck E Shields JG Gordon J Smith SH Boyd A Callard RE. Recombinant human interleukin 5 is an eosinophil differentiation factor but has no activity in standard human B cell growth factor assays. Eur J Immunol. (1987) 17:1743–50. doi: 10.1002/eji.1830171210 3500861

[78] Clutterbuck EJ Hirst EM Sanderson CJ. Human interleukin-5 (IL-5) regulates the production of eosinophils in human bone marrow cultures: comparison and interaction with IL-1, IL-3, IL-6, and GMCSF. Blood. (1989) 73:1504–12. doi: 10.1182/blood.V73.6.1504.bloodjournal7361504 2653458

[79] Greenfeder S Umland SP Cuss FM Chapman RW Egan RW. Th2 cytokines and asthma. The role of interleukin-5 in allergic eosinophilic disease. Respir Res. (2001) 2:71–9. doi: 10.1186/rr41 PMC59571 11686868

[80] Hirai K Yamaguchi M Misaki Y Takaishi T Ohta K Morita Y. Enhancement of human basophil histamine release by interleukin 5. J Exp Med. (1990) 172:1525–8. doi: 10.1084/jem.172.5.1525 PMC2188680 1700056

[81] Resnick MB Weller PF. Mechanisms of eosinophil recruitment. Am J Respir Cell Mol Biol. (1993) 8:349–55. doi: 10.1165/ajrcmb/8.4.349 8476627

[82] She S Ren L Chen P Wang M Chen D Wang Y. Functional roles of chemokine receptor CCR2 and its ligands in liver disease. Front Immunol. (2022) 13:812431. doi: 10.3389/fimmu.2022.812431 35281057 PMC8913720

[83] Zlotnik A Yoshie O. The chemokine superfamily revisited. Immunity. (2012) 36:705–16. doi: 10.1016/j.immuni.2012.05.008 PMC3396424 22633458

[84] Carr MW Roth SJ Luther E Rose SS Springer TA. Monocyte chemoattractant protein 1 acts as a T-lymphocyte chemoattractant. Proc Natl Acad Sci USA. (1994) 91:3652–6. doi: 10.1073/pnas.91.9.3652 PMC43639 8170963

[85] Ranjbar M Rahimi A Baghernejadan Z Ghorbani A Khorramdelazad H. Role of CCL2/CCR2 axis in the pathogenesis of COVID-19 and possible Treatments: All options on the Table. Int Immunopharmacol. (2022) 113:109325. doi: 10.1016/j.intimp.2022.109325 36252475 PMC9561120

[86] Shibuya R Ishida Y Hanakawa S Kataoka TR Takeuchi Y Murata T. CCL2-CCR2 signaling in the skin drives surfactant-induced irritant contact dermatitis through IL-1beta-Mediated neutrophil accumulation. J Invest Dermatol. (2022) 142:571–582 e9. doi: 10.1016/j.jid.2021.07.182 34560074

[87] Jimenez F Quinones MP Martinez HG Estrada CA Clark K Garavito E. CCR2 plays a critical role in dendritic cell maturation: possible role of CCL2 and NF-kappa B. J Immunol. (2010) 184:5571–81. doi: 10.4049/jimmunol.0803494 PMC2929965 20404272

[88] Isozaki T Amin MA Ruth JH Campbell PL Tsou PS Ha CM. Fucosyltransferase 1 mediates angiogenesis, cell adhesion and rheumatoid arthritis synovial tissue fibroblast proliferation. Arthritis Res Ther. (2014) 16:R28. doi: 10.1186/ar4456 24467809 PMC3978694

[89] Wang Z Hu J Pan Y Shan Y Jiang L Qi X. miR-140-5p/miR-149 affects chondrocyte proliferation, apoptosis, and autophagy by targeting FUT1 in osteoarthritis. Inflammation. (2018) 41:959–71. doi: 10.1007/s10753-018-0750-6 29488053

[90] Amin MA Campbell PL Ruth JH Isozaki T Rabquer BJ Alex Stinson W. A key role for Fut1-regulated angiogenesis and ICAM-1 expression in K/BxN arthritis. Ann Rheum Dis. (2015) 74:1459–66. doi: 10.1136/annrheumdis-2013-204814 24665114

[91] Moore GT Brown SJ Winterhalter AC Lust M Salvaris EJ Selan C. Glycosylation changes in hFUT1 transgenic mice increase TCR signaling and apoptosis resulting in thymocyte maturation arrest. Mol Immunol. (2008) 45:2401–10. doi: 10.1016/j.molimm.2007.11.006 18155296

[92] Kaneko M Nishihara S Shinya N Kudo T Iwasaki H Seno T. Wide variety of point mutations in the H gene of Bombay and para-Bombay individuals that inactivate H enzyme. Blood. (1997) 90:839–49. doi: 10.1182/blood.V90.2.839.839_839_849 9226185

[93] Kelly RJ Ernst LK Larsen RD Bryant JG Robinson JS Lowe JB. Molecular basis for H blood group deficiency in Bombay (Oh) and para-Bombay individuals. Proc Natl Acad Sci USA. (1994) 91:5843–7. doi: 10.1073/pnas.91.13.5843 PMC44093 7912436

[94] Scharberg EA Olsen C Bugert P. The H blood group system. Immunohematology. (2016) 32:112–8. doi: 10.21307/immunohematology-2019-056 27834485

[95] Jajosky RP Wu SC Zheng L Jajosky AN Jajosky PG Josephson CD. ABO blood group antigens and differential glycan expression: Perspective on the evolution of common human enzyme deficiencies. iScience. (2023) 26:105798. doi: 10.1016/j.isci.2022.105798 36691627 PMC9860303

