# Supplementary figures and images for: Impact of fucosyltransferase 1-mediated epidermal blood group antigen H on anti-inflammatory response in atopic dermatitis
Source: Front Immunol. 2024 May 22;15:1365430. doi: 10.3389/fimmu.2024.1365430 (PMC11151169; doi:10.3389/fimmu.2024.1365430)

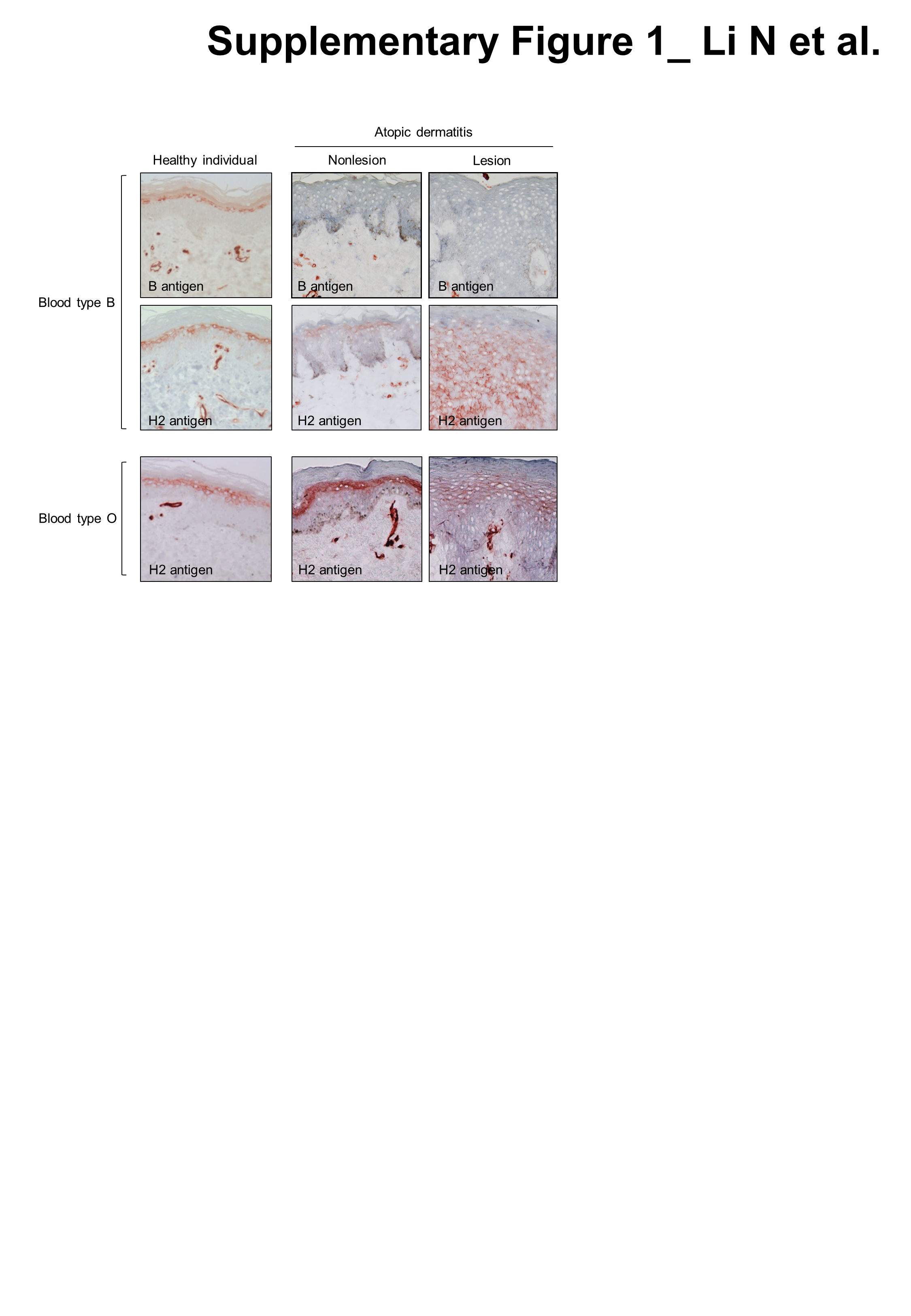

Supplement: Supplementary Figure 1 — The granular layers of healthy skin exhibit ABH antigen expression which is diminished in nonlesional and lesional AD skin. Immunohistochemical staining to detect B and H antigens in healthy skin, as well as on nonlesion and lesioned AD skin from individuals with blood types B or O. AD, atopic dermatitis. [file Image_1.tif]

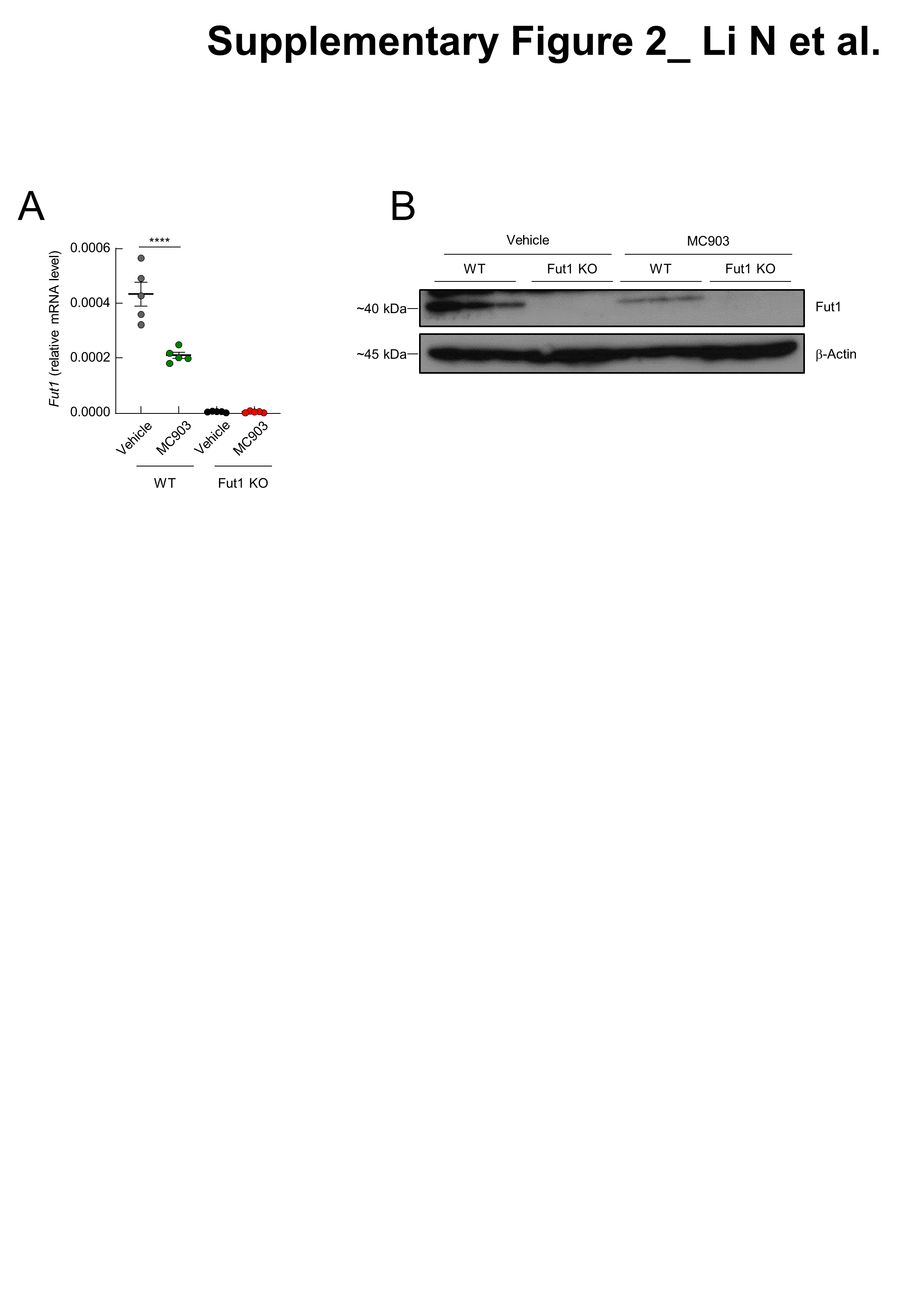

Supplement: Supplementary Figure 2 — Reduced expression levels of Fut1 mRNA and protein in the skin of WT mice treated with MC903. (A) Relative mRNA expression of Fut1 in the ear skin of both WT and Fut1-KO mice treated with vehicle or MC903 on day 12. (B) The protein expression of Fut1 and β-Actin in the ear skin of both WT and Fut1-KO mice treated with vehicle or MC903 on day 12. The data represent the mean ± SEM of three independent experiments, with five mice per group. ns., not significant; **** p < 0.0001. [file Image_2.tif]

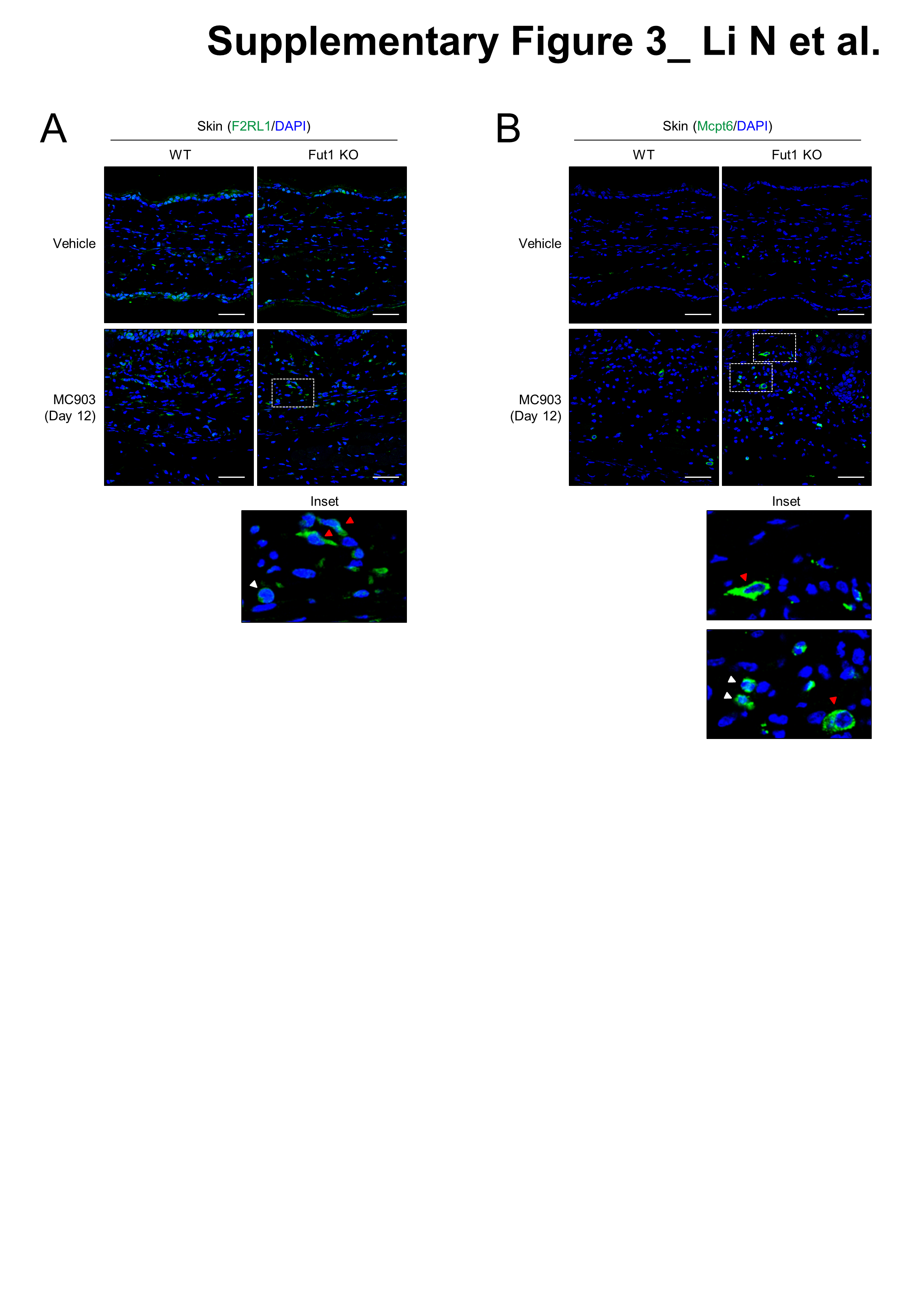

Supplement: Supplementary Figure 3 — Expression of mast cell activation markers in the skin. (A) Immunofluorescent labeling of F2RL1 (green) and DAPI (blue) in ear sections on day 12. (B) Immunofluorescent labeling of Mcpt6 (green) and DAPI (blue) in ear sections on day 12. [file Image_3.tif]
